# Iron deficiency anemia in pregnancy and related complications with specific insight in Rivers State, Nigeria: a narrative review

**DOI:** 10.1097/MS9.0000000000003224

**Published:** 2025-04-02

**Authors:** Getrude Uzoma Obeagu, Basil Omieibi Altraide, Emmanuel Ifeanyi Obeagu

**Affiliations:** a Department of Midwifery and Child Health Nursing, University of Port Harcourt, Rivers State, Nigeria; b Department of Obstetrics and Gynaecology, Rivers State University Teaching Hospital, Port Harcourt, Nigeria; c Department of Biomedical and Laboratory Science, Africa University, Mutare, Zimbabwe

**Keywords:** anemia, complications, iron deficiency, pregnancy, red blood cell indices

## Abstract

Iron deficiency anemia (IDA) is one of the most common nutritional disorders affecting pregnant women worldwide, with significant implications for maternal and neonatal health. In developing regions like Rivers State, Nigeria, the prevalence of IDA in pregnancy remains alarmingly high, exacerbated by factors such as poverty, inadequate healthcare access, suboptimal nutrition, and endemic parasitic infections like malaria. This narrative review provides an updated overview of IDA in pregnancy, emphasizing its prevalence, risk factors, and complications, with a specific focus on Rivers State. The review highlights the multifaceted consequences of IDA, including maternal outcomes such as increased risks of preeclampsia, postpartum hemorrhage, and mortality, alongside fetal complications like intrauterine growth restriction, low birth weight, and perinatal mortality. Current interventions, including antenatal iron and folic acid supplementation programs, have achieved limited success due to logistical challenges, low health literacy, and cultural barriers in the region. To address these issues effectively, a multipronged approach is essential, involving community-based health education, improved access to affordable healthcare services, and policy-driven efforts to address systemic barriers. This review emphasizes the urgent need for improved strategies to mitigate the burden of IDA in Rivers State and similar settings, ultimately improving pregnancy outcomes and advancing maternal and child health.

## Introduction

Iron deficiency anemia (IDA) in pregnancy is a critical public health concern worldwide, with profound implications for maternal and neonatal health. Anemia during pregnancy is defined as a hemoglobin concentration below 11 g/dL and is predominantly caused by iron deficiency, which accounts for over half of all cases globally^[1]^. IDA is the most common type of anemia in pregnancy apart from hemodilution^[2]^. Iron deficiency is usually caused by inadequate dietary iron intake, and is considered the most common nutritional deficiency leading to anemia. Deficiencies of vitamin A, folate, vitamin B12, and riboflavin can also cause anemia due to their specific roles in the synthesis of hemoglobin and/or erythrocyte production^[3]^. IDA was defined by the American College of Obstetricians and Gynecologists with a hemoglobin of 10.0 g/dL or higher as well as a low or normal mean corpuscular volume (MCV) of 80–100 fL^[1]^. Also,^[3]^ World Health Organization estimated that 50% of the anemia seen in pregnancy is due to iron deficiency and defined anemia in pregnancy as a condition characterized by low level of red blood cells (RBCs) or hemoglobin content of blood with hemoglobin concentration less than 11 g/dL in first trimester, less than 10.5 g/dL in second trimester, and less than 11 g/dL in third trimester. World Health Organization further classified it as mild anemia for hemoglobin value of 9–10.9 g/dL, moderate anemia with hemoglobin value of 7–8.9 g/dL and severe anemia where hemoglobin value is lower than 7 g/dL^[3]^. Low MCV signifies low hemoglobin, iron deficiency and microcytic anemia^[4]^. High level of MCV also known as macrocytosis, is linked mainly to vitamin B12 and folic acid deficiency or other pathologic conditions^[5]^.

**HIGHLIGHTS**

- Common condition: Iron deficiency anemia affects 15%–25% of pregnancies worldwide.
- Symptoms: Causes fatigue, dizziness, and breathlessness.
- Risks: Linked to preterm birth, low birth weight, and postpartum hemorrhage.
- Diagnosis: Identified via hemoglobin and serum ferritin tests.
- Treatment: Managed with iron supplements, dietary changes, or IV iron therapy in severe cases.

IDA during pregnancy places women at risk for poor pregnancy outcomes leading to maternal mortality, perinatal mortality, premature birth, and low birth weight. Infants born to anemic mothers have significantly very low iron reserves. Iron deficiency is also associated with reduced work capacity and reduced neurocognitive development^[6]^. World Health Organization recommends daily iron of 30–60 mg and folic acid 400 µg (0.4 mg) supplementation in pregnant women throughout pregnancy beginning as early as possible for pregnant adolescents and adult women in all healthcare setting. In places where anemia in pregnant women is a severe public health problem up to 40% higher, a daily dose of 60 mg of elemental iron is recommended^[6]^. Pregnant woman diagnosed with anemia in a clinical setting, daily iron 120 mg and folic acid 400 µg or 0.4 mg supplementation should be given until her hemoglobin level rises to normal and continued with the standard antenatal dose to prevent recurrence of anemia^[6]^. In addition to iron and folic acid, other vitamins and minerals may be given to overcome other maternal micronutrient deficiencies. In malaria-endemic areas, provision of iron and folic acid supplements should be implemented together with measures to prevent, diagnose and treat malaria^[7]^. An iron and folic acid supplementation program should ideally form part of an integrated program of antenatal and neonatal care that promotes healthy pregnancy outcome^[7]^. Prevention and early detection with clinical manifestations of breathing difficulties, fainting, tiredness, palpitations plus sleep difficulties as well as proper diagnosis and effective treatment of IDA depend on availability of professional midwives as well as other members of health professional team and obtainability of iron/folic acid in the health facility at affordable price for the pregnant mothers^[8]^. In most rural health centers, health professionals, equipment, drug and both governmental as well as nongovernmental agencies support are lacking^[9]^. Pregnant mothers in the rural areas prefer to depend on traditional birth attendants for their pregnancy care^[10]^. Also, the environment of the pregnant women, occupation, and nutrition have impact on health of the mother as well as developing fetus. In area like Rivers State, South South, Nigeria where there are oil mill activities, there is usually air pollution, water pollution, and oil spillage on farmlands. Human life especially pregnant mother with their developing fetus is adversely affected by heavy metals, chemicals, and their toxic effect in human body^[11]^. The heavy metals like lead and magnesium occupy the bone marrow displacing erythroid tissue and interfere with erythropoiesis leading to decrease as well as ineffective erythropoiesis^[12,13]^. Heavy metal like lead exposure from water pollution is a risk factor to IDA in humans. Iron deficient increases lead absorption which can cause lead toxicity. Iron and lead interact and compete in heme synthesis^[14]^. Nigeria, as the most populous country in Africa, bears a significant portion of the global burden of IDA in pregnancy^[15]^. The situation is particularly terrible in Rivers State, located in the Niger Delta region, where socio-economic challenges, poor dietary habits, and endemic diseases like malaria exacerbate the problem. Understanding the multifaceted nature of IDA in pregnancy requires a comprehensive analysis of its epidemiology, causes, and consequences^[16]^. This review of iron deficiency in pregnancy and associated complications with region-specific insight becomes necessary to identify its rate of occurrence and health consequences in Rivers State.

## Prevalence of IDA in pregnancy

Iron is a critical nutrient during pregnancy due to its essential role in supporting the increased blood volume and oxygen transport required for both the mother and developing fetus^[17]^. The prevalence of IDA during pregnancy is a critical public health issue, especially in rural areas where access to healthcare and nutritional resources might be limited^[18]^. The prevalence of iron deficiency in pregnancy refers to the proportion of pregnant women who have inadequate iron levels, which can lead to IDA if not addressed^[19]^. Understanding this prevalence is crucial for public health planning and intervention, especially in rural areas with limited healthcare resources. Iron deficiency is one of the most common nutritional deficiencies during pregnancy and can have significant impacts on both maternal and fetal health^[3]^. Globally, IDA affects approximately 36.5% of pregnant women worldwide, according to the World Health Organization^[20]^. Internationally, anemia is estimated to affect half a billion women 15–49 years of age and 269 million children 6–59 months of age worldwide. In 2019, 30% (539 million) of nonpregnant women and 37% (32 million) of pregnant women aged 15–49 years were affected by anemia^[3]^. Africa and Southeast Asia are most affected with an estimated 106 million women and 103 million children affected by anemia in Africa and 244 million women and 83 million children affected in Southeast Asia^[3]^. The largest causes were dietary iron deficiency, thalassemia, and sickle cell trait^[3]^. The World Health Organization has recognized IDA as the most common nutritional deficiency in the world, with 30% of the population being affected with this condition^[18]^.

The prevalence varies widely by region, with higher rates in low- and middle-income countries due to factors such as poor dietary intake, higher rates of infections, and limited access to healthcare^[21]^. In regional variations involving some regions of Africa and Southeast Asia, the prevalence of anemia in pregnant women can be as high as 60%–70%^[21]^. In rural areas of countries like India, studies have reported prevalence rates of IDA in pregnant women to be around 50%–70%^[22]^. In high-income countries, Australia as well as the United States of America, the prevalence is lower but still significant, affecting about 20% and 18% in pregnant women^[23,24]^. Studies showed that the prevalence of IDA in pregnant women in rural areas can be significantly higher than in urban areas. In some rural regions, rates of anemia among pregnant women can reach 32.9%^[25]^. Anemia caused 50 million years of healthy life lost due to disability in 2019 and the largest cause was dietary iron deficiency^[3]^. World Health Organization reports that almost two billion people or 25% of the world’s population are anemic, with roughly half of them having IDA^[20,26]^. WHO reported there are no current global data for iron deficiency, using anemia as an indirect indicator^[20]^. World Health Organization estimated that most pregnant women in developing countries and at least 30%–40% in developed countries, are iron deficient. Nearly half of the pregnant women in the world are estimated to be anemic; 52% in developing countries compared with 23% in developed countries^[20]^.

In developed countries, most pregnant women are thought to suffer from some degree of iron deficiency. Anemia is particularly prominent in South Asia; in India, up to 88% of pregnant and 74% of nonpregnant women are affected^[20]^. In Africa, about 50% of pregnant and 40% of nonpregnant women are anemic. West Africa is the most affected, and Southern Africa the least. In Latin America as well as the Caribbean, occurrence of anemia in pregnant women was 40% and nonpregnant was 30%^[20]^. In Nigeria, the prevalence of IDA among pregnant women is notably high, reflecting a broader trend across the country^[27]^. In systematic review of IDA in pregnancy reported it is highly prevalent among pregnant women in Nigeria, with rates ranging from 25% to 45.6%. IDA in pregnancy was influenced by factors such as low socioeconomic status, multiparity, and late-stage pregnancy^[27]^. Nutritional deficiencies, inadequate healthcare access, poor utilization of antenatal care services, higher rates of infections such as malaria and intestinal parasites exacerbate the situation^[28]^.

Reported prevalence of iron deficiency 41% among pregnant women in Lagos and Kano states North West, Nigeria with moderate or severe anemia and its prevalence increased with gestational age occurring among 4 in 10 pregnant women. In Southwest Nigeria,^[29]^ testified 47.3% anemia prevalence for nonpregnant women aged 15–49 years and pregnant women was 57.5%. Specifically, IDA was reported in 25%–46% of pregnant women with anemia

In Southeast Nigeria^[30]^, reported prevalence of iron deficiency 40.8%, IDA 23.6%, and non-IDA 24.7% while about two out of every 5 (40.8%) had serum ferritin level less than 15 μg/L. Further concluded high prevalence of IDA among the respondents in both trimesters even with intake of recommended oral iron supplements. In Rivers State, South South, Nigeria^[31]^, found prevalence of anemia in pregnancy 86.4% but did not specify prevalence of IDA^[32]^. Carried out a study on prevalence of anemia in pregnancy in rural and urban areas of Akwa Ibom State, South South, Nigeria and reported 61.1% prevalence of anemia in pregnancy, with 10.2% mild anemia and 50.9% moderate anemia while the average daily dietary intake of iron was 17.7 mg (6.1–37.8 mg). Also, in Cross River State, South South, Nigeria, the prevalence of IDA was significantly higher 32.0% as well as 76.0% among pregnant women in two rural hospitals while their Serum iron (ferritin) reported no significant difference^[33]^. There was no study found on the prevalence of IDA in pregnancy in Rivers State, South South, Nigeria.

## Diagnosis of IDA in pregnancy

The diagnosis of IDA during pregnancy involves a combination of clinical evaluation, laboratory testing, and assessment of risk factors^[34]^. Clinical presentation of IDA in pregnancy includes symptoms such as fatigue, weakness, shortness of breath, dizziness or lightheadedness, palpitations, and pallor noticeable on the skin, conjunctiva, and nail beds^[35]^. However, symptoms can overlap with normal pregnancy changes, laboratory tests are crucial for diagnosis. Laboratory testing is complete blood count (CBC); hemoglobin (Hb), MCV and mean corpuscular hemoglobin (MCH). Hemoglobin (Hb) is a protein found in RBCs that is essential for the transport of oxygen and carbon dioxide throughout the body^[36]^. World Health Organization defines anemia in pregnancy as Hb <11 g/dL. IDA is suspected if Hb levels are low, particularly <11 g/dL in first trimester, <10.5 g/dL in second trimester, and <11 g/dL in the third trimester^[37]^. Hemoglobin levels were classified based on the World Health Organization criteria for anemia in pregnancy, mild is Hb of 10.0–10.9 g/dL, moderate: 7.0–9.9 g/dL, and severe: <7.0 g/dL^[38]^.

MCV is a measure used in hematology to describe the average size of RBCs (erythrocytes) in a blood sample^[4]^. It is a key parameter in a CBC test and is useful in diagnosing different types of anemia and other blood disorders. The normal range of MCV during pregnancy is generally similar to that of nonpregnant adults, approximately 80–100 femtoliters (fL)^[4]^. MCV may increase slightly in pregnancy due to physiological changes, including the increased production of RBCs and plasma volume expansion. Macrocytosis (MCV >100 fL) can occur due to folate or vitamin B12 deficiency, which are more common during pregnancy. Low MCV (<80 fL) suggests microcytic anemia, commonly caused by iron deficiency or thalassemia. High MCV (>100 fL) suggests macrocytic anemia, often related to folate or vitamin B12 deficiency. Normal MCV (80–100 fL) can be seen in normocytic anemia for example, anemia of chronic disease or blood loss^[4]^. Regular monitoring of MCV, along with hemoglobin, hematocrit, and ferritin levels, is crucial for identifying and addressing anemia during pregnancy to ensure maternal and fetal health. MCH is the average amount of hemoglobin (Hb) contained in a single RBC. The normal range of MCH during pregnancy is generally similar to that of nonpregnant adults, typically 27–33 picograms (pg) per cell^[39]^. MCH values may remain stable during pregnancy if maternal iron, folate, and vitamin B12 levels are adequate. Physiological changes in blood volume during pregnancy (hemodilution) can impact other blood parameters but usually do not significantly affect MCH^[40]^. Low MCH (<27 pg), indicates hypochromic anemia, commonly caused by IDA, which is prevalent during pregnancy due to increased iron demands for fetal development. High MCH (>33 pg), indicates macrocytic anemia or hyperchromic anemia^[39]^. Usually associated with folate or vitamin B12 deficiency, which can occur due to higher nutritional demands during pregnancy.

Serum ferritin is a protein that stores and releases iron, making it the most reliable indicator of iron status and body iron stores^[41]^. In pregnancy, its interpretation is crucial due to the increased demand for iron to support fetal growth, placental development, and maternal blood volume expansion^[17]^. It acts as an iron storage protein in cells and releases iron when needed. Serum ferritin reflects total body iron stores under normal conditions. Serum ferritin <30 µg/L is highly suggestive of iron deficiency during pregnancy. Levels <15 µg/L indicates depleted iron stores^[42,43]^. Serum iron measures the amount of circulating iron bound to transferrin, and it reflects the immediate availability of iron for metabolic processes^[44]^. The normal reference range for serum iron levels in women is 50–170 µg/dL (9–30 µmol/L)^[45]^. During pregnancy, serum iron levels tend to decrease due to increased iron demand, hemodilution, and hormonal changes^[43]^. Normal serum iron in pregnancy is 30–150 µg/dL (5.4–26.9 µmol/L)^[46]^. Serum iron levels alone are insufficient for diagnosing IDA in pregnancy, as they can fluctuate based on recent dietary intake and diurnal variation. Serum ferritin, TIBC, and transferrin saturation provide more reliable diagnostic insights for assessing iron status. The normal range for total iron binding capacity (TIBC) in nonpregnant females is 240–450 µg/dL (43–81 µmol/L)^[47]^. During pregnancy, the normal range for TIBC is higher due to increased transferrin production, which facilitates iron transport to meet the growing demands of the fetus^[48]^. Normal TIBC in Pregnancy is 250–450 µg/dL (45–81 µmol/L). TIBC tends to be on the higher end of the range during pregnancy compared to nonpregnant women due to physiological adaptations. Elevated TIBC (>450 µg/dL) is commonly seen in IDA, reflecting increased transferrin production in response to low iron stores. Lower values (<240 µg/dL) can occur in conditions such as chronic diseases, hemochromatosis, or malnutrition. TIBC represents the blood’s capacity to bind iron with transferrin^[48]^. TIBC is used in conjunction with other iron studies (serum iron, ferritin, transferrin saturation) to assess iron status and differentiate types of anemia.

During pregnancy, transferrin saturation reflects the proportion of transferrin that is bound with iron, which is essential for evaluating iron status^[48]^. Due to the physiological changes of pregnancy, such as increased plasma volume and iron demands, normal ranges may vary slightly. Normal range in pregnancy is 20%–45% in first trimester, and 16%–45% in second and third trimesters^[49]^. Transferrin saturation <16% is considered low and indicative of IDA in pregnancy reflecting insufficient iron availability for erythropoiesis, consistent with IDA^[48]^. Elevated transferrin saturation may occur in conditions like iron overload or hemochromatosis but is rare in pregnancy^[49]^. In pregnancy, lower transferrin saturation should be evaluated with other iron studies, such as serum ferritin and total iron-binding capacity, to confirm the diagnosis of IDA^[50]^. In addition, peripheral blood smear indicating microcytic, hypochromic red blood cells. Reticulocyte Count may be low or normal in IDA despite anemia^[51]^. Differential diagnosis of IDA from other causes of anemia in pregnancy, such as physiological anemia of pregnancy caused by hemodilution, has normal iron levels with mild anemia. Vitamin B12 or folate deficiency called macrocytic anemia characterized by high MCV. Thalassemia which may mimic IDA but is identified by hemoglobin electrophoresis or genetic testing^[52]^. Risk factors for IDA in pregnancy were inadequate dietary iron intake, pre-existing anemia or heavy menstrual bleeding, short inter-pregnancy interval, multiple gestations, history of gastrointestinal disorders (e.g. malabsorption syndromes), and vegetarian or vegan diet without supplementation^[53]^.

## Maternal complication associated with IDA in pregnancy

IDA during pregnancy can cause several complications due to its impact on both the mother and the developing fetus^[54]^. The primary issues stem from reduced oxygen-carrying capacity of the blood as well as decreased blood volume and subsequent effects on maternal and fetal health. Hemoglobin, which contains iron, is essential for transporting oxygen from the lungs to tissues. IDA reduces hemoglobin levels, leading to hypoxia (low oxygen levels) in maternal and fetal tissues^[55]^. The fetus receives less oxygen and nutrients which potentially impaired growth and development. To compensate for low oxygen levels, the heart pumps more blood, increasing cardiac output. This can lead to cardiovascular strain and complications such as gestational hypertension and preeclampsia^[56]^. However, specific pregnancy complications associated with IDA on mother are preterm labor, gestational hypertension and preeclampsia, postpartum hemorrhage (PPH), increased fatigue and weakness as well as infections^[57]^. Preterm labor, defined as labor that begins before 37 weeks of gestation, is a significant concern during pregnancy. IDA can increase the risk of preterm labor due to various physiological stresses and changes in the maternal body^[58]^. IDA leads to a decrease in hemoglobin levels, reducing the blood’s ability to carry oxygen to maternal and fetal tissues^[55]^. The fetus relies on the mother’s blood for oxygen. Hypoxia can stimulate the release of stress hormones, which may trigger preterm labor. Hypoxia can cause increased uterine activity and contractions, potentially leading to preterm labor. Hypoxia can increase the release of prostaglandins, which are chemicals that promote uterine contractions^[59]^.

IDA affects the placenta’s ability to function properly, leading to placental insufficiency^[54]^. This can reduce nutrient and oxygen supply to the fetus, creating a hostile environment that can induce preterm labor. The placenta itself may become hypoxic due to reduced oxygen levels in maternal blood, contributing to its dysfunction and the release of stress signals that initiate labor prematurely. Hypoxia induces cellular stress within the placental tissues, disrupting various cellular processes necessary for maintaining placental health and functionality. Hypoxic placental cells release various stress signals and inflammatory mediators, including cytokines and hormones, which can affect the surrounding maternal and fetal tissues^[60]^. These stress signals can stimulate uterine contractions and cervical changes, potentially initiating labor prematurely. The body may perceive the hypoxic environment as hostile, triggering mechanisms to expel the fetus earlier than the full term to ensure survival. IDA weakens the immune system, making pregnant women more prone to infections. Infections can cause inflammation. Infections and inflammation can lead to the release of cytokines, which can weaken the fetal membranes and increase the risk of their rupture, leading to preterm labor^[18]^. The body may prioritize blood flow to vital organs, reducing uterine blood flow and contributing to uterine hypoxia and irritability causing preterm labor^[61]^. In physiological stress and maternal body response, the maternal body responds to hypoxia and anemia by activating stress pathways. These stress responses can lead to the production of hormones and other factors that can induce labor. Corticotropin-releasing hormone, released in response to stress, can stimulate the production of prostaglandins and promote uterine contractions leading to preterm labor^[62]^. Understanding these mechanisms highlights the importance of early detection and management of IDA in pregnant women to reduce the risk of preterm labor and improve pregnancy outcomes.

IDA during pregnancy can contribute to the development of gestational hypertension and preeclampsia through several interconnected mechanisms. IDA reduces the hemoglobin levels and consequently decreases the blood’s oxygen-carrying capacity^[55]^. This triggers the body’s compensatory mechanisms to increase blood volume, which can strain the cardiovascular system and lead to hypertension. Iron is essential for the synthesis of nitric oxide (NO), a vasodilator that helps regulate blood vessel tone. IDA-induced deficiency in NO production may lead to vasoconstriction and increased vascular resistance, contributing to hypertension^[63]^. IDA is associated with increased oxidative stress due to impaired antioxidant defenses. Oxidative stress can damage the endothelial cells lining blood vessels, leading to inflammation and dysfunction. The endothelial dysfunction caused by oxidative stress can disrupt the normal regulation of vascular tone and increase the risk of hypertension and preeclampsia^[64]^. IDA compromises placental function by reducing oxygen delivery to the placenta. This can lead to hypoxia and oxidative stress within the placenta, triggering a cascade of events that contribute to hypertension and preeclampsia^[60]^. IDA alters the balance of angiogenic factors such as vascular endothelial growth factor and soluble tyrosine kinase-1. Disruption in this balance is implicated in the pathogenesis of preeclampsia^[65]^. IDA alters hormone levels, including those involved in blood pressure regulation and fluid balance. These hormonal changes can predispose to hypertension and preeclampsia^[66]^. Understanding these mechanisms highlights the importance of addressing and managing IDA early in pregnancy to mitigate the risk of gestational hypertension and preeclampsia. Regular prenatal care, including screening for anemia, nutritional interventions, and iron supplementation, is essential in preventing these serious pregnancy complications.

IDA during pregnancy has been associated with an increased risk of gestational diabetes mellitus. IDA can disrupt hormonal regulation, including insulin sensitivity^[67]^. IDA leads to increased oxidative stress and inflammation. Oxidative stress can impair insulin signaling pathways, leading to reduced insulin sensitivity. Inflammation, marked by elevated levels of cytokines like TNF-alpha and IL-6, can also interfere with insulin signaling and contribute to insulin resistance^[68]^. Iron is crucial for mitochondrial function and energy production. In IDA, reduced iron availability impairs mitochondrial function, leading to decreased ATP production and altered glucose metabolism^[69]^. This impairment can result in decreased insulin sensitivity as cells struggle to efficiently utilize glucose. Iron deficiency can alter the function of adipose tissue, affecting the secretion of adipokines like leptin and adiponectin. Adiponectin is particularly important for maintaining insulin sensitivity^[70]^. Insulin resistance, a key factor in the development of gestational diabetes mellitus, may be exacerbated in women with IDA. Reduced levels of adiponectin in IDA can lead to increased insulin resistance^[71]^. IDA can cause tissue hypoxia (reduced oxygen levels) because of decreased hemoglobin and oxygen transport capacity. Hypoxia-inducible factors are activated under low oxygen conditions and can negatively affect insulin signaling pathways, contributing to insulin resistance^[72]^. Iron plays a role in glucose metabolism and insulin function. Insufficient iron levels may lead to altered glucose metabolism, potentially contributing to insulin resistance and gestational diabetes mellitus. IDA can compromise placental function, affecting nutrient and oxygen transfer to the fetus. Placental dysfunction is linked to metabolic disturbances, including insulin resistance and gestational diabetes mellitus^[73]^.

Early screening and diagnosis of IDA during pregnancy are crucial. Iron supplementation and dietary interventions can help improve iron status and potentially reduce the risk of gestational diabetes mellitus^[74]^. Monitoring blood glucose levels and managing insulin resistance through lifestyle modifications (diet and exercise) and, if necessary, pharmacological interventions are key to preventing complications. IDA during pregnancy has been associated with an increased likelihood of cesarean section (C-section). IDA often leads to maternal fatigue and weakness due to reduced oxygen-carrying capacity in the blood^[55]^. Fatigue can impact the woman’s ability to tolerate the physical demands of labor, potentially leading to interventions such as C-sections for maternal exhaustion. IDA can impair placental function, potentially leading to complications such as intrauterine growth restriction (IUGR) or fetal distress^[75]^. These complications may necessitate a C-section for the well-being of the baby. IDA is associated with an increased risk of preterm labor. In cases where preterm delivery is indicated to protect the health of the baby, a C-section may be performed. Women with IDA are at higher risk of PPH due to impaired blood clotting mechanisms^[76]^. In cases where there is concern for excessive bleeding, a C-section may be preferred to manage potential complications. Individualized care and timely interventions are crucial in managing pregnancies complicated by IDA to minimize the need for C-sections when possible.

IDA can contribute to PPH, which is defined as excessive bleeding (>500 mL after vaginal delivery or >1000 mL after cesarean delivery) within the first 24 hours following childbirth^[76]^. IDA decreases the amount of hemoglobin available to carry oxygen, but it also affects other aspects of blood function, including clotting factors^[55]^. This can impair the blood’s ability to form clots effectively, leading to prolonged bleeding after childbirth. Iron is necessary for proper platelet function. Platelets are crucial in forming blood clots to stop bleeding^[76]^. In IDA, platelet function can be impaired, leading to inadequate clot formation and increased bleeding tendency. IDA during pregnancy can contribute to uterine atony, which is a significant cause of PPH^[77]^. Uterine atony occurs when the uterus fails to contract effectively after childbirth, leading to excessive bleeding. IDA decreases hemoglobin levels, which compromises the amount of oxygen delivered to tissues, including uterine muscle tissues^[55]^. This can lead to muscle weakness throughout the body, including the uterus. Anemia-related muscle weakness may exacerbate uterine atony, leading to increased bleeding. Iron is essential for proper muscle function, including the contraction and relaxation of uterine muscles during labor and delivery^[78]^. Inadequate iron levels can impair these functions, potentially leading to ineffective uterine contractions and uterine atony^[79]^. In compensatory mechanisms where the body compensates for reduced oxygen-carrying capacity, the body may increase blood volume during pregnancy (hemodilution)^[80]^. This increased blood volume can contribute to higher volumes of blood loss postpartum in women with IDA^[76]^. IDA is associated with impaired coagulation, muscle weakness, delayed uterine involution, and increased blood volume advocating the importance of addressing and managing IDA to minimize the risk of PPH and ensure maternal well-being after childbirth^[81]^.

IDA during pregnancy can increase the risk of infections through various mechanisms related to immune function and overall health. Iron is essential for the proper functioning of the immune system^[82]^. IDA compromises the immune response by reducing the activity of immune cells, impairing their ability to recognize and fight off pathogens effectively^[83]^. Antibodies, also known as immunoglobulins, are proteins produced by the immune system in response to specific pathogens (like bacteria or viruses). They recognize and bind to these pathogens, marking them for destruction by other immune cells. Iron is necessary for the development and function of immune cells, including B lymphocytes that produce antibodies^[84]^. In IDA, the production and activity of B lymphocytes may be impaired due to insufficient iron, leading to reduced antibody production. Lower levels of antibodies mean that the body may have a diminished ability to recognize and neutralize pathogens effectively^[84]^. This can increase susceptibility to infections and prolong the duration of illnesses. Cytokines are small proteins that act as signaling molecules in the immune system. They regulate inflammation, immune cell activation, and communication between cells during an immune response^[85]^. Iron deficiency can disrupt the production and function of cytokines^[86]^. For example, IDA may alter the balance between pro-inflammatory cytokines (which promote inflammation and immune responses) and anti-inflammatory cytokines (which regulate and resolve inflammation). Impaired regulation of cytokine production can impact the body’s ability to mount an appropriate immune response. This may lead to exaggerated inflammatory responses or inadequate immune activation against pathogens, both of which can contribute to increased susceptibility to infections^[83]^. Understanding how IDA affects antibody production and cytokine production provides insights into its impact on immune function. Addressing IDA through appropriate management and supplementation is critical to support a robust immune response and reduce susceptibility to infections during pregnancy and postpartum.

IDA during pregnancy can contribute to prolonged labor through several mechanisms related to maternal health and uterine function. IDA decreases hemoglobin levels, leading to reduced oxygen-carrying capacity in the blood. This can result in muscle weakness and fatigue, including the muscles of the uterus^[55]^. Iron is crucial for proper muscle function, including uterine muscle contractions during labor^[81]^. Insufficient iron levels may impair the strength and frequency of uterine contractions, potentially prolonging labor. IDA weakens the immune system, making women more susceptible to infections such as urinary tract infections or chorioamnionitis. Infections can lead to inflammation and uterine dysfunction, contributing to prolonged labor^[83]^. Iron deficiency can affect hormonal regulation, including oxytocin levels. Oxytocin is a hormone produced by the hypothalamus and released by the pituitary gland. It plays a key role in uterine contractions during labor and delivery, as well as in milk ejection during breastfeeding^[87]^. Oxytocin is a hormone that stimulates uterine contractions. Imbalances in oxytocin production or response due to IDA may contribute to prolonged labor^[88]^. Iron is a critical cofactor for enzymes involved in the synthesis and metabolism of various hormones, including oxytocin^[89]^. Oxytocin is synthesized in the hypothalamus as a precursor peptide, and its final active form is processed and released from the posterior pituitary gland. Insufficient iron levels can disrupt these enzymatic processes, potentially impairing the proper synthesis and release of oxytocin^[90]^. This disruption may lead to reduced levels of oxytocin available for stimulating uterine contractions during labor^[88]^.

Iron deficiency can disrupt the normal functioning of the hypothalamic-pituitary axis. This disruption may affect the regulatory mechanisms that control oxytocin synthesis, storage, and release^[91]^. As a result, there may be alterations in the timing, amplitude, or duration of oxytocin release during labor. This could affect the coordination and strength of uterine contractions necessary for effective labor progression. Iron deficiency can also affect the duration of oxytocin release. Prolonged or inadequate release of oxytocin may contribute to prolonged labor, where contractions are insufficient to achieve cervical dilation and fetal descent^[91]^. IDA has been linked to an increased risk of postpartum depression (PPD) and the exact mechanisms are complex^[92,93]^. Iron is essential for the synthesis and metabolism of neurotransmitters like serotonin and dopamine, which play key roles in mood regulation. Insufficient iron levels can lead to alterations in neurotransmitter function, potentially contributing to depressive symptoms^[94]^. IDA often causes fatigue and decreased energy levels, which can exacerbate the physical and emotional challenges of early motherhood. Persistent fatigue can contribute to feelings of overwhelm and sadness, common in PPD. Iron deficiency can affect thyroid function, leading to hormonal imbalances that may influence mood regulation. Thyroid hormones play a role in neurotransmitter metabolism and can affect overall mental health^[91]^. IDA can influence the body’s stress response system, particularly affecting cortisol regulation, which plays a crucial role in managing stress and mood^[91]^. Cortisol is a hormone produced by the adrenal glands in response to stress. It helps regulate metabolism, immune response, and the body’s response to stressors. Iron is involved in the synthesis and regulation of cortisol^[95]^. Insufficient iron levels can disrupt the normal production and release of cortisol, leading to dysregulation in its levels. In some cases of IDA, the body may produce higher levels of cortisol as a compensatory mechanism to manage stress. This can lead to chronic stress responses, contributing to feelings of anxiety, irritability, and mood swings^[96]^.

IDA may also lead to decreased cortisol levels in other individuals. Low cortisol levels can impair the body’s ability to respond to stress effectively, potentially exacerbating feelings of fatigue, lethargy, and emotional instability. Dysregulated cortisol levels have been associated with mood disorders, including depression. Both high and low cortisol levels due to IDA can contribute to changes in mood, energy levels, and overall mental well-being^[96]^. IDA can contribute to delayed wound healing through several physiological mechanisms related to impaired immune function and collagen synthesis. Iron is essential for the proper functioning of the immune system^[83]^. IDA compromises immune function by reducing the activity of immune cells involved in wound healing, such as neutrophils and macrophages^[97]^. IDA weakens the immune response, making individuals more susceptible to infections. Infections at the wound site can delay the healing process and lead to complications. Iron is a cofactor for enzymes involved in collagen synthesis, which is essential for tissue repair and wound closure. Insufficient iron levels can impair collagen production, leading to delayed wound healing^[98]^. Inadequate collagen synthesis due to IDA may result in poor-quality scar tissue formation, further delaying the healing process and increasing the risk of wound complications^[98]^. Iron is a component of hemoglobin, the protein responsible for transporting oxygen from the lungs to tissues throughout the body. IDA reduces hemoglobin levels, limiting oxygen delivery to the wound site^[55]^. Reduced oxygen supply (hypoxia) at the wound site can impair cellular metabolism and function, slowing down the healing process and prolonging recovery^[99]^. Managing IDA and promoting optimal wound healing involves treating the underlying iron deficiency and supporting the body’s healing processes. IDA is associated with impaired immune function, reduced collagen synthesis, and compromised oxygen delivery leading to delayed wound healing and overall health^[99]^. Globally, studies have shown complications associated with IDA in pregnancy. In British Columbia, 12.8% of respondents had anemia in pregnancy. Anemia was associated with preterm birth 1.09%, small-for-gestational-age live birth 2.26%, and neonatal death, and perinatal death 2.27%^[100]^. In Texas Medical Center USA, the prevalence of anemia was 35.8% among pregnant women. Successfully treated anemic patients with oral iron therapy had preterm birth 5.1% and preeclampsia 5.9%. Not responding to treatment and untreated patients had increased rates of preterm birth 8.3% and preeclampsia 8.3%^[101]^. In Egypt, prevalence of anemia in pregnancy was 42%. Majority of cases were IDA 92.8% and sickle cell trait, B-thalassemia intermedia, and other causes 7.2% despite iron supplementation. PPH was 5.4%, cesarean delivery 40.3%, and infections 3.8%^[102]^. There was no study conducted in Nigeria on complications associated with IDA in pregnancy.

## Fetal complications of IDA in pregnancy

Iron is essential for fetal growth and brain development. Iron deficiency during pregnancy is associated with preterm birth, low birth weight, and impaired cognitive and motor development in infants^[17]^. Adequate maternal iron levels help ensure sufficient iron stores in newborns, which are critical for their growth and development in the first 6 months of life^[103]^. Insufficient oxygen and nutrients can lead to IUGR, resulting in low birth weight^[104]^. Babies born to anemic mothers are more likely to be anemic themselves, which can affect their development and health. Chronic hypoxia and nutrient deficiencies can lead to developmental delays and cognitive impairments in the child^[105]^. Iron plays a crucial role in various aspects of placental function, which is essential for the proper development and well-being of the fetus^[106]^. IDA can impact placental function and contribute to complications such as fetal growth restriction (FGR)^[75]^. The placenta serves as a vital interface between the maternal and fetal circulations, facilitating the transfer of nutrients (including iron) and oxygen from the mother to the developing fetus^[107]^. Iron is particularly important for the synthesis of hemoglobin and myoglobin, which are essential for oxygen transport. Iron is actively transported across the placenta to meet the fetal demand for growth and development^[106]^. In cases of IDA, this transport mechanism may be compromised, leading to inadequate iron supply to the fetus^[54]^. Iron deficiency can reduce maternal hemoglobin levels, leading to maternal anemia. This reduces the oxygen-carrying capacity of maternal blood, potentially impairing oxygen delivery to the placenta and fetus. Insufficient iron availability due to IDA can restrict fetal growth and development^[108–110]^. The fetus may not receive adequate nutrients and oxygen needed for optimal growth, leading to FGR or IUGR^[17]^. IDA-related placental dysfunction and FGR can increase the risk of preterm birth, where the baby is born before completing 37 weeks of gestation^[75]^.

In severe cases, IDA-induced FGR may have long-term consequences on neurodevelopment and overall health outcomes of the child. IDA’s impact on maternal hemoglobin levels and iron transport can contribute to complications such as FGR and preterm birth^[17]^. Managing IDA during pregnancy is critical to optimize placental function, support fetal growth, and reduce the risk of adverse pregnancy outcomes. Public health initiatives, including education, routine screening, and accessible prenatal care, are essential in treating IDA, especially in rural and underserved areas^[111–116]^. In Central China, the prevalence of anemia in early pregnancy was 16.3%. The severity of maternal anemia was associated with preterm birth 1.37% for mild anemia, moderate anemia 1.54%, and severe anemia 4.03%. Low birth weight was mild anemia 1.61%, moderate anemia 2.01%, and severe anemia 6.11%. Small for gestational age for mild anemia was 1.37%, moderate anemia was 1.54% and severe anemia was 2.61%^[110]^. In Egypt, Prevalence of anemia in pregnancy was 42%. Majority of cases were IDA 92.8% and sickle cell trait, B-thalassemia intermedia, and other causes 7.2% despite iron supplementation. The prevalence of Low Apgar score was 11.8%, preterm birth 12.9% and low birth weight babies were 11.3%^[102]^.

## Conclusion

IDA remains a critical challenge during pregnancy in Rivers State, Nigeria, with significant maternal and neonatal health implications. Managing this health issue requires a multifaceted approach, including improving nutrition, strengthening healthcare services, and addressing socio-economic determinants. Enhanced focus on localized interventions and continued research are essential for reducing the burden of IDA in this region.

## Data Availability

Not applicable as this is a review.

## Research registration unique identifying number (UIN)

Not applicable as this is a review.
